# Unusual cause of dyspnoea: a case presentation of an echocardiographic pitfall

**DOI:** 10.1186/1749-8090-8-230

**Published:** 2013-12-17

**Authors:** Carina Primus, Gernot Grabscheit, Choi Keung Ng, Johann Auer

**Affiliations:** 1 Department of Cardiology and Intensive Care, General Hospital Braunau, Ringstrasse 60, Braunau A - 5280, Austria; 2 Cardiothoracic surgeon in Innsbruck (A 6020), Museumstrasse 33/1, Tirol, Austria

**Keywords:** Dyspnoea, Pulmonary hypertension, Gerbode defect

## Abstract

Congenital or acquired communication between the left ventricle and the right atrium is known as the Gerbode defect, which is rarely diagnosed since the defect is very unusual and for this reason often misinterpreted as an eccentric tricuspid regurgitation jet.

The entity and reason of the defect is unknown to many physicians, so that profound knowledge and a careful and meticulous echocardiogram are necessary in order to prevent misinterpretation of this defect as a pulmonary hypertension.

We report the case of a 76-year-old Austrian woman who developed such a Gerbode defect after a recent bioprosthetic aortic valve replacement.

## Background

The causes for new occurrence of dyspnoea are manifold. However, especially cardiologists are confronted with echocardiographic findings more and more often, which are not common in routine daily work – on the one hand due to the increasing age of the population and the associated increasingly recurring cardiac defects which often require surgery and on the other hand due to successfully remediated congenital cardiac defects originated in the early childhood with now normal expectancy of life.

We report the case of a patient who early after aortic valve replacement felt recurring dyspnoea and showed signs of cardiac decompensation.

## Case presentation

A 76-year-old Austrian woman with recent bioprosthetic aortic valve replacement presented herself with recurring progressive dyspnoea (NYHA III).

She had been operated half a year ago because of a hemodynamically relevant stenosis of the tricuspid aortic valve. Access was enabled by mediane sternotomy and after application of the bypass (entire duration of an hour) and initiation of cardioplegia the calcificated valve was replaced by a mitroflow pericardial prothesis with a diameter of 21 mm. Operation was proceeded without any complications and the patient got extubated already the day after.

After initial improvement of symptoms within the first months she finally again suffered from a rapid drop of her performance level; however, she did not observe any other general symptoms or fever. The patient clinically showed signs of cardiac decompensation with minimal pretibial edema, but without any orthopnoea. An electrocardiogram showed an already known atrial fibrillation in normal frequency without pathological signs of repolarisation.

Auscultation, however, revealed a 3/6 holosystolic murmur at the left parasternal border with multidirectional conduction, so that again a two-dimensional transthoracic echocardiography was performed. Apart from normal chamber sizes and left ventricular (LV) ejection fraction, a huge dilatation especially of the right atrium with low tricuspid regurgitation due to coaptation dysfunction was shown with a systolic regurgitation velocity of 2.8 m/sec. which equates to a slight pulmonal hypertension.

**Figure 1:**
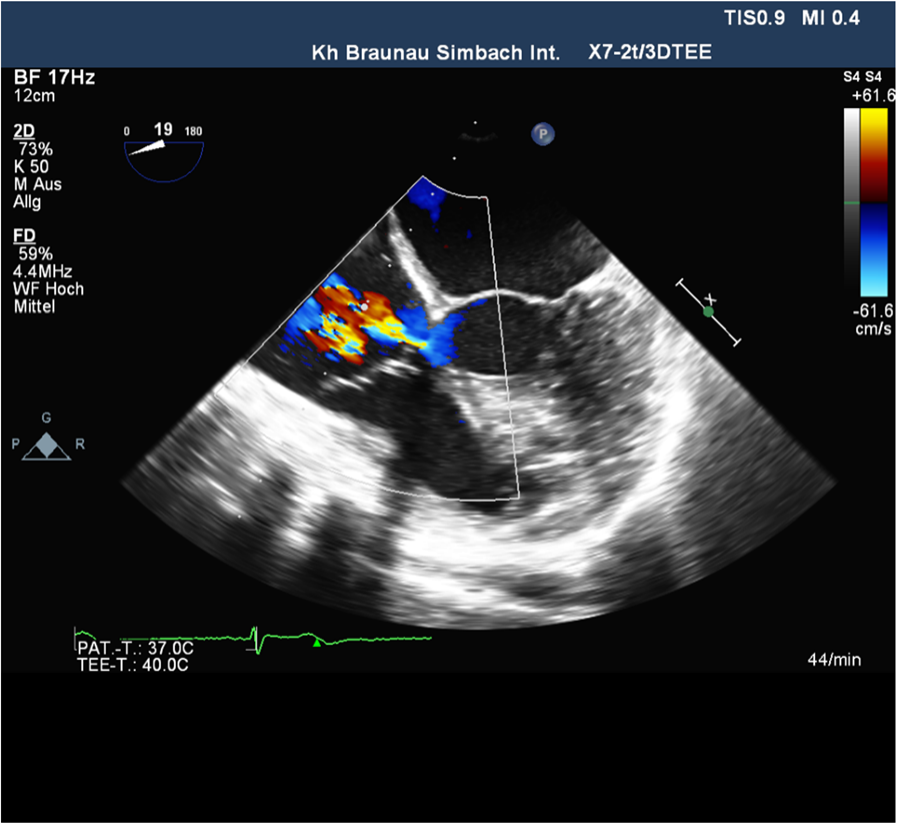
Two-dimensional transesophageal echocardiogram shows the Gerbode defect (arrows) in apical 4-chamber view.

**Figure 2:**
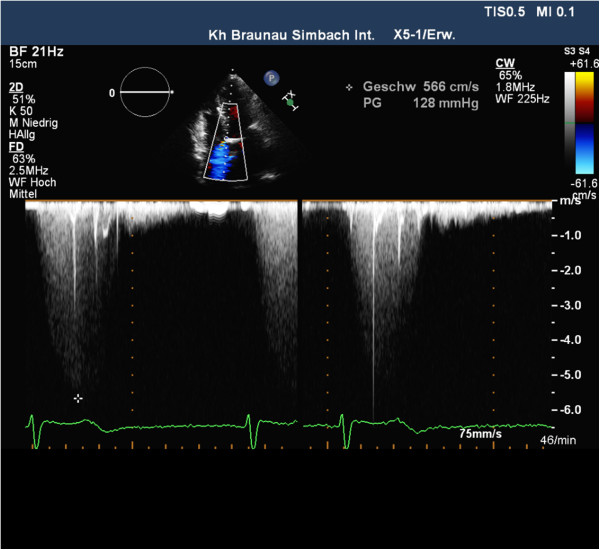
Two-dimensional color-flow Doppler echocardiogram shows blood flow during systole from the left ventricle to the right atrium through the Gerbode defect (arrows) in apical 4-chamber view.

Additionally, however, a high-velocity systolic jet just below the aortic valve was noticed entering the right atrium (RA), which was initially mistaken for tricuspid regurgitation (Figure 1). Spectral Doppler distinguished a velocity of 5.6 m/sec, whereas a three-dimensional transesophageal echocardiography confirmed the jet taking a direct course from the left ventricle (LV) into the RA, consistent with a Gerbode defect (Figure 2).

Due to the lack of typical symptoms of an endocarditis and the newly occurring pulmonary hypertension as well as the LV-RA-shunt, the recent aortic valve replacement was finally assumed to be the reason for this.

As a result diuretic therapy was increased and drug therapy optimized which led to an improvement of the patient’s condition (NYHA II). The Gerbode defect finally required no surgical intervention.

## Conclusion

A LV-to-RA communication is a perimembranous defect of either congenital cause or the result of valvular surgery, blunt trauma, infective endocarditis, myocardial infarction, and postsurgical repair of ventricular septal defects (VSD). It was first classified in 1958 by Gerbode as a very rare congenital defect [1].

All perimembranous defects are beneath the aortic valve. The membranous septum is separated into two parts according to the insertion of the septal leaflet of the tricuspid valve: one atrioventricular and one interventricular component.

Due to the different level of insertion of the two AV-valves a part of the membranous septum as also the inlet septum separates the left ventricle from the right atrium. Defects in this area can lead to a VSD of the AV-canal or, as in our case report, to a rare LV-RA-shunt, the Gerbode defect.

While the congenital defect is often associated with tricuspid valve abnormities and lies interventricular respectively directly under the insertion of the septal leaflet of the tricuspid valve, the shunt above the tricuspid valve (atrioventricular) typically points to an acquired form [2].

The actual cause of an acquired Gerbode defect is still unclear. It is supposed for defects originating from endocarditis due to a bacterial infection (most often Staphylococcus aureus) at the subannular region crossing over to the membranous septum and thus causing a LV-RA shunt [3].

Early after valve replacement the Gerbode defect can be related both to the extension of the valvular debridement and to the erosion of the membranous septum by the rigid prosthetic ring. Although subaortic membrane is detected easily by echocardiographic examination, a Gerbode defect might be misinterpreted erroneously as a severe pulmonary hypertension. Shunting across a Gerbode defect mainly occurs in systole since LV systolic pressure is much higher than RA pressure and it often appears to be a high velocity jet in spectral Doppler.

As soon as the physician notices the combination of a huge right atrium with only a low tricuspid valve regurgitation and a critical eccentric jet above the tricuspid valve, a possible Gerbode defect should thoroughly be taken into account and special attention should be drawn to the membranous septum.

If this cannot be excluded echocardiographically, a transesophageal echocardiography should be performed for further evaluation.

Moreover, a cardiac MRI can be provided in order to detect the anatomy and the hemodynamics in detail and to be able to plan the further procedure regarding a possible repair by surgery or a catheter-based intervention.

For longer periods a Gerbode defect can result in congestive (right) heart failure and exercise intolerance according to pronounced dyspnoea, arrhythmias and cardiac decompensation. For this reason it is mostly recommended to perform surgery on this defect.

Catheter-based intervention is an upcoming procedure to close particularly the iatrogen Gerbode defect avoiding anew open-heart surgery showing indeed good results until now.

Surgical repair, however, is still the common approach to close the defect by a simple direct suture or an insertion of a patch (autologous or bovine) requiring cardiopulmonary bypass and cardioplegic diastolic arrest to provide a bloodless, motionless field for intracardiac closure.

Potential surgical complications include infection, postoperative bleeding requiring re-exploration, valve injury (tricuspid, pulmonary, or aortic), pulmonary hypertension with poor cardiac output, AV-blockage and other severe arrhythmias, and death. The overall surgical mortality rate is indeed low, as long as risk factors such as relevant comorbidities, the patient’s age and other associated cardiac anomalies don’t exist.

In case of the reported patient only after the recent aortic valve replacement we resigned from anew intervention, since she was advanced in years and resilient again after cardiac recompensation.

Especially the new onset of the tricuspid regurgitation and the jet of an indeed medium-sized LV-RA-shunt still overloading the right heart as well as the diastolic disorder of relaxation were believed to be the reason of the patient’s dyspnoea and further cardiac decompensation.

**Figure 3:**
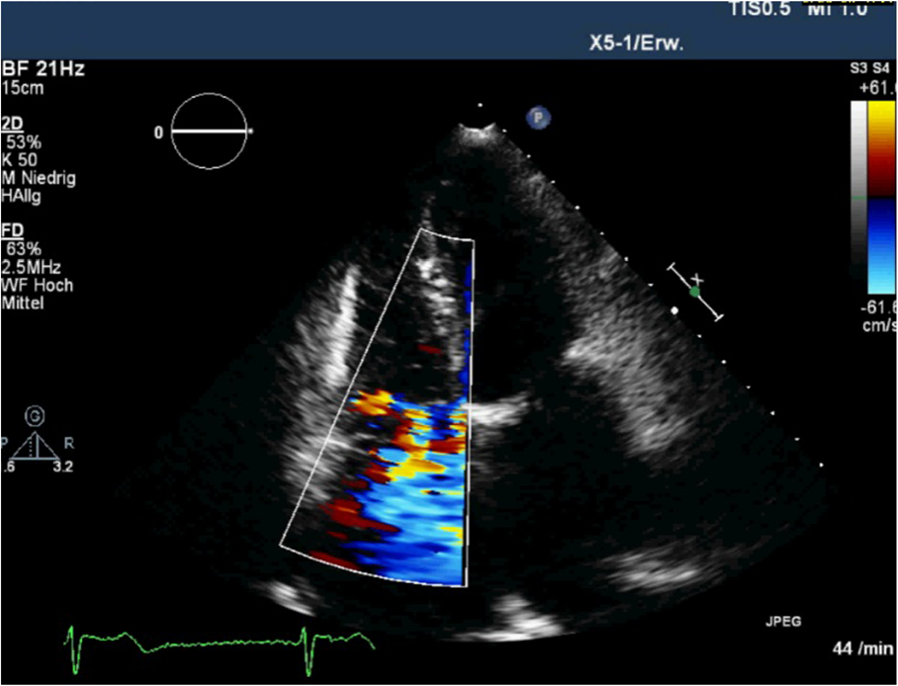
Two-dimensional color-flow Doppler echocardiogram shows the Gerbode defect as well as the low tricuspid regurgitation (arrows) in apical 4-chamber view.

After having optimized medication she finally hardly sensed dysponea anymore and afterwards reported a general satisfaction. Also, the right and the left ventricle had normal size and the pulmonary hypertension was only low so that according to the guidelines of the American Heart Association no further interventions were indicated (Figure 3).

In agreement with the patient continuous clinical and echocardiographic check-ups are carried out now in order to be able to detect the hemodynamic activity of the defect in time.

In summary, the Gerbode defect is a rare complication, it might be still misinterpreted as only pulmonary hypertension and therefore be underdiagnosed.

New onset of dyspnoea after cardiac surgery should always lead to further investigation, whereas transthoracic and – esophageal echocardiography are convenient diagnostic instruments in addition to a simple auscultation.

Besides a loud holosystolic murmur a huge right atrium with an only low tricuspid valve regurgitation and a critical eccentric jet above the tricuspid valve are seminal.

For longer periods a Gerbode defect can result in congestive (right) heart failure and further complications, wherefore careful anamnesis, examination and profound knowledge in echocardiography are necessary in order to diagnose it and to be able to plan the further procedure regarding a possible repair by (re-) surgery or a catheter-based intervention.

## Consent

Written informed consent was obtained from the patient for publication of this case report and accompanying images. A copy of the written consent is available for review by the Editor-in-Chief of this journal.

## Abbreviations

NYHA: New York Heart Association; LV: Left ventricular; RV: Right ventricular; RA: Right atrium/atrial; VSD: Ventricular septal defect; AV: Atrioventricular; MRI: Magnet resonance imaging.

## Competing interests

The authors declare that they have no competing interests.

## Authors’ contributions

CP was the main author and wrote the article. GG did the echocardiogram. NC was the cardiothoracic expert and surgical advisor. JA was the cardiology consultant and gave final approval of the manuscript. All authors have read and approved the final manuscript.
